# Nonepisodic angioedema with eosinophilia after COVID-19 vaccination: a case successfully treated with reslizumab

**DOI:** 10.1186/s13223-023-00765-8

**Published:** 2023-02-02

**Authors:** Young-Hee Nam

**Affiliations:** Department of Internal Medicine, Dong-A University College of Medicine, Busan, Korea

**Keywords:** Angioedema, Eosinophilia, COVID-19, Vaccines, Reslizumab

## Abstract

**Background:**

Angioedema with eosinophilia (AE) is a rare allergic disease classified as episodic or nonepisodic. AE is characterized by angioedema, urticaria, fever, weight gain, and eosinophilia, but its etiology and pathogenesis have not yet been clarified.

**Case presentations:**

We present a 70-year-old woman presented with generalized edema and urticaria after Moderna COVID-19 vaccination. Peripheral blood eosinophil count was marked elevated and echocardiography and Doppler ultrasonography of both the upper and lower extremities were unremarkable. Her symptoms and peripheral blood eosinophil count were improved after systemic steroid therapy, but she failed to respond to steroid tapering. Reslizumab (anti-interluekin-5) was administered intravenously, and she remained symptom free with a normal eosinophil count during 8 months of reslizumab treatment without steroids.

**Conclusions:**

We report a case of nonepisodic AE after COVID-19 vaccination that was successfully treated with reslizumab.

## Introduction

As of 24 November 2022, 13 billion doses of coronavirus disease 2019 (COVID-19) vaccine have been administered globally, and 68.5% of the world’s population has received at least one dose of vaccine in the era of the pandemic COVID-19 [1]. Various adverse reactions to vaccines, such as systemic, neurologic, cardiovascular, hematologic, and cutaneous reactions, have been reported [2]. Cutaneous reactions to COVID-19 vaccines are relatively mild and self-limited [3].

Angioedema with eosinophilia (AE) is a rare allergic disease but its etiology and pathogenesis have been unclear. We describe a case of AE after COVID-19 vaccination with steroid-dependency that was successfully treated with reslizumab, an anti-interluekin-5 (IL-5) antibody. Written informed consent was obtained from the patient.

## Case

**Fig. 1:**
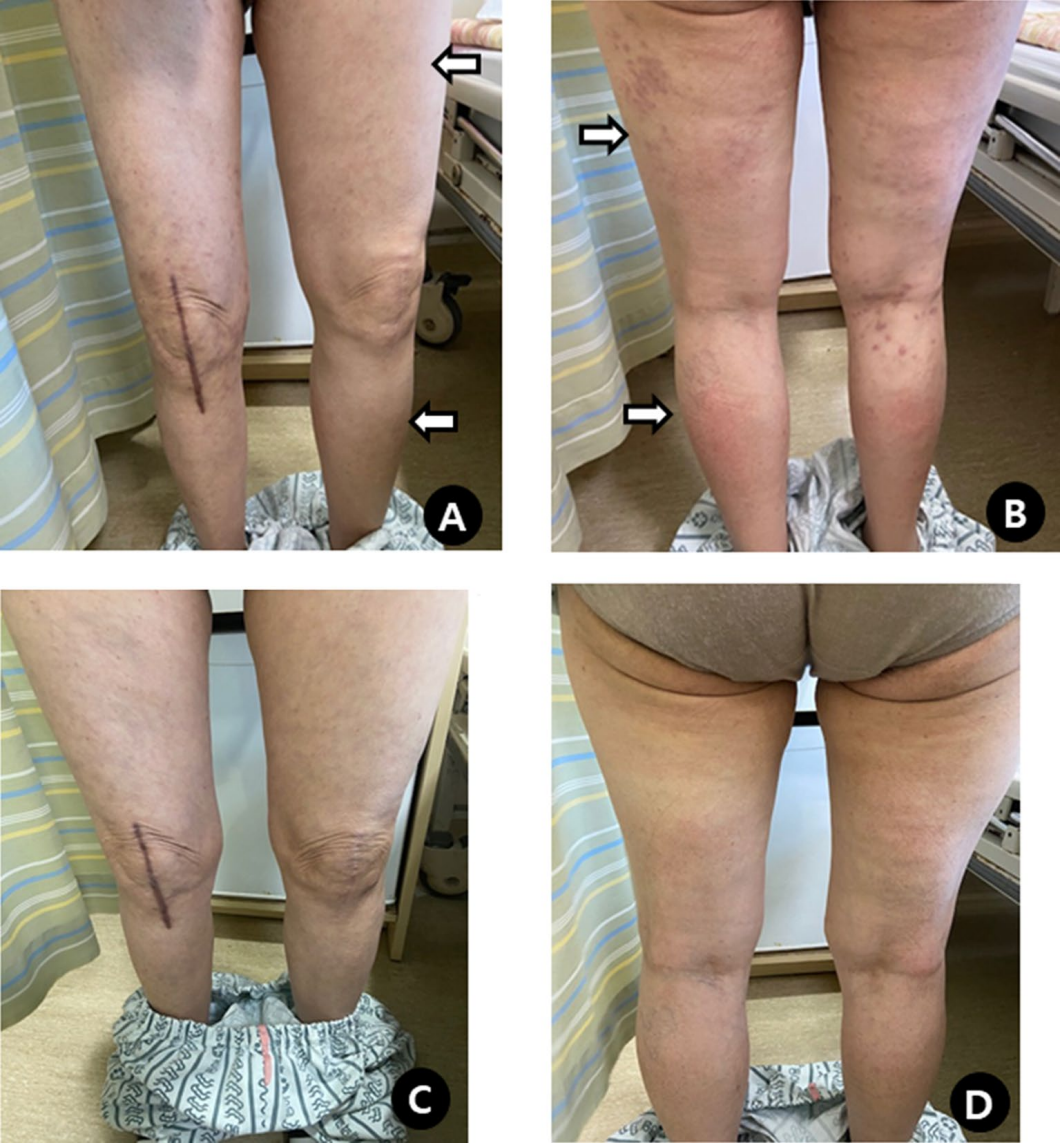
Non-pitting edema of both lower extremities (**A** and **B**), and which disappeared after steroid treatment (**C** and **D**)

A 70-year-old woman presented with generalized edema and urticaria 5 days after the first Moderna vaccination (the 3rd dose of COVID-19 vaccine). She had a medical history of diabetes mellitus. There were no adverse reactions after the previous 1st and 2nd doses of Oxford-AstraZeneca vaccination. Edema firstly developed in the left upper arm at the injection site and then spread to both extremities and trunk, especially on the left side (Fig. 1A and B), and she gained 10 kg body weight. Urticaria was also observed in both the arms and legs.

**Fig. 2:**
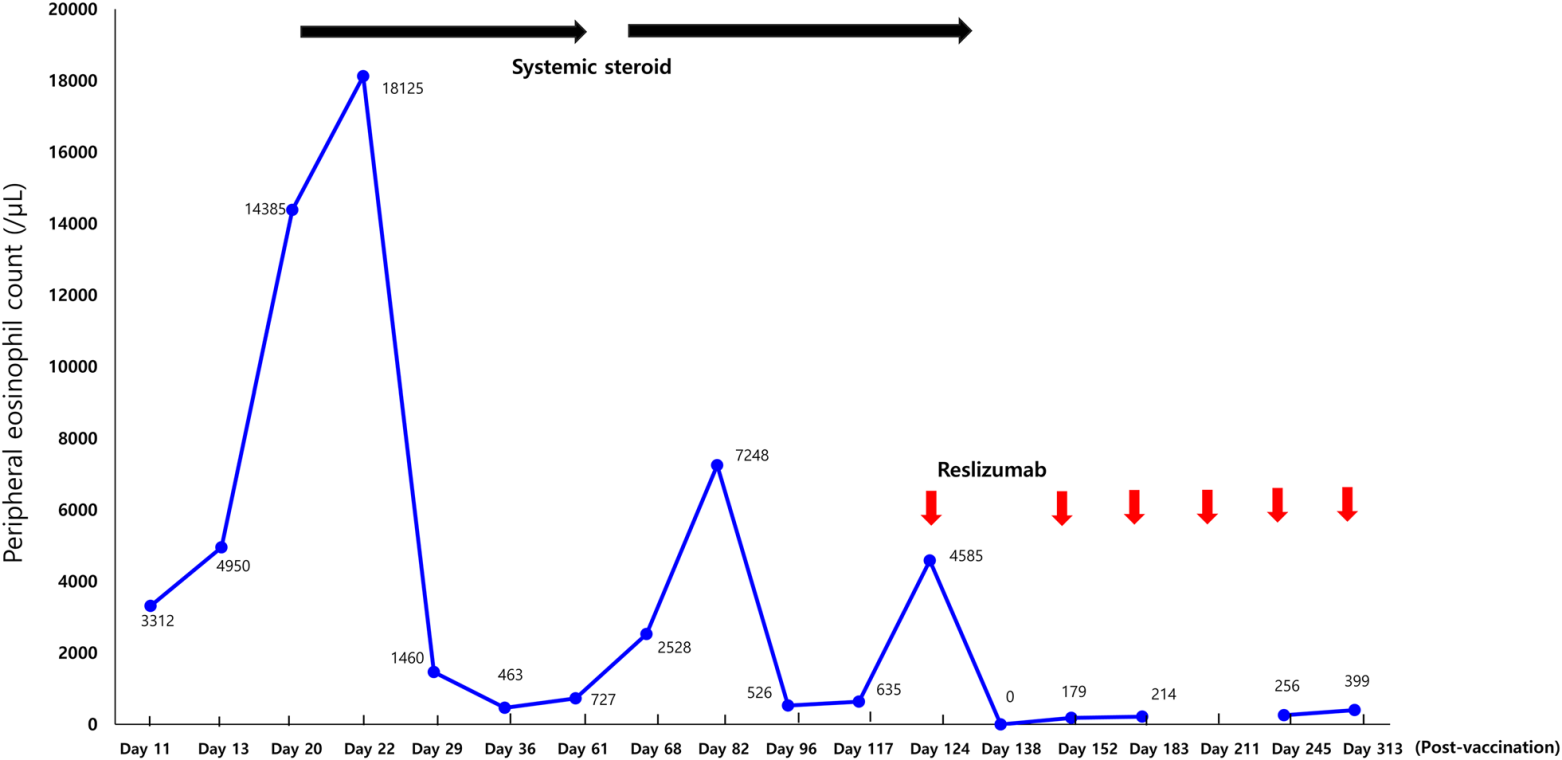
Change of peripheral eosinophil count after systemic steroid (black arrows) and reslizumab (red arrows) therapy

Laboratory tests showed an elevated peripheral blood eosinophil count (3,312/µL), which increased to 18,125/µL. Serum total immunoglobulin (Ig) E (293 IU/mL) was slightly elevated, and IgM (122.4 mg/dL) was within the normal range. C1 esterase inhibitor, C1 inactivator activity, C4, anti-nuclear antibody, and stool analysis results were normal or negative. There were no genetic abnormalities, including *JAK2, CALR, MPL, FIP1L1-PDGFR A*, and *BCR-ABL1*. Echocardiography, chest computed tomography, abdominal ultrasonography, and Doppler ultrasonography of both the upper and lower extremities were unremarkable. Intravenous steroids (methylprednisolone 1 mg/kg/day) were administered to the patient for 7 days. Urticaria resolved rapidly, whereas angioedema improved gradually and disappeared completely (Fig. 1 C and D). Oral prednisolone was tapered off over 5 weeks. The blood eosinophil counts became normal. Seven days after steroid discontinuation, the blood eosinophil count increased (2,528/µL) and angioedema affecting the face and upper limbs recurred. Oral prednisolone was restarted and tapered from 30 to 5 mg/day over 4 weeks. On the 5th day of maintaining 5 mg/day of prednisolone, laryngeal edema developed, and the blood eosinophil count increased to 7.248/µL. Reslizumab 200 mg (3 mg/kg) was administered every 4 weeks along with prednisolone (30 mg/day for 7 days, 20 mg/day for the next 7 days) for the first 14 days. At the 3rd injection, reslizumab was tapered to 100 mg and maintained at the same dose for 4 weeks, and the 6th infusion was done at 8-week intervals. (Fig. 2). She remained symptom free with a normal eosinophil count during 8 months of reslizumab treatment without steroids.

## Discussion

AE is classified as episodic (EAE) or nonepisodic (NEAE). EAE first described in 1976 [4] is characterized by recurrent episodes of angioedema, urticaria, fever, weight gain, elevated peripheral blood eosinophil counts, and elevated serum IgM levels [5]. NAEA has been mainly reported in Korea and Japan [6, 7], has a less severe clinical course than EAE, and is suggested to be a milder form of EAE [7–9]. NEAE is characterized by a single episode of edema of the extremities, arthralgia, eosinophilia, and normal IgM levels [8]. The pathogenesis of AE has not yet been clarified. Activated T-cell-derived cytokines, mainly IL-5, may be involved in the activation of blood and tissue eosinophils that drive AE [5, 10]. Serum IL-5 and total IgE concentrations were parallel with peripheral blood eosinophilia and clinical symptoms, and related with disease activity [11]. Eosinophilic degranulation releases proinflammatory cytokines, including IL-5, chemokines, and eosinophil-specific granules, such as major basic protein, eosinophil cationic protein, and eosinophil-derived neurotoxin, which are associated with inflammatory reactions and edema [12]. Another study suggested that multiple lineages other than eosinophils, including lymphocytes, neutrophils and mast cells, are involved in AE pathogenesis [13]. However, the triggers of these immunologic reactions, and whether these cells act or promote eosinophil activation, have not been elucidated. Systemic corticosteroids have been widely administered for the management AE owing to their suppressive effects on eosinophil and Th2 cytokines.

A wide range of adverse reactions to COVID-19 vaccines have been reported. Inflammatory cytokines, autoimmune involvement, angiotensin-converting enzyme 2 downregulation, and eosinophil association have been suggested to be related to post-vaccine adverse reactions [14]. Eosinophilic diseases after COVID-19 vaccination included eosinophilic myocarditis [15, 16], eosinophilic pneumonia [16–18], eosinophilic granulomatosis with polyangiitis [19], eosinophilic cellulitis [20, 21], eosinophilic panniculitis [22], eosinophilic gastroenteritis [23] and NEAE [24]. Eosinophil responses are observed not only during COVID-19 infection but also during coronavirus vaccination [25]. Whether eosinophils have a protective or exacerbating role during COVID-19 remains unclear. Eosinopenia was noted in patients with COVID-19, and it may be a prognostic factor for COVID-19 severity. A Th2-skewed immunopathology was observed, and isolated coronavirus spike protein caused eosinophilia and eosinophil infiltration in the lungs associated with a Th2 response in severe acute respiratory syndrome coronavirus 1 (SARS-CoV-1) murine vaccine studies [26–28]. The SARS-CoV-2 spike protein of COVID-19 can also lead to eosinophil activation owing to the sharing of high identity between SARS-CoV-1 and SARS-CoV-2 [25].

To date, only one case of AE after COVID-19 vaccination has been reported [24]. The patient developed NEAE after the 2nd dose of Pfizer-BioNTech vaccination and showed a good response to systemic steroids. The patient in this case developed NEAE after Moderna vaccination (previous 1st and 2nd Oxford-AstraZeneca vaccines). Many conditions can cause eosinophilia, and a comprehensive approach is needed for an accurate diagnosis. Moreover, the diagnostic criteria for AE is not yet well defined, it remains one of exclusion. Although it was not perfect, a thorough evaluation was conducted for this patient. She failed to respond to steroid tapering, but her symptoms and blood eosinophilia were well-controlled after reslizumab administration. Anti-IL-5 therapy is indicated for eosinophilic diseases, such as asthma, eosinophilic granulomatosis with polyangiitis, and eosinophilic chronic rhinosinusitis, and blood eosinophil count is the best-established biomarker for the prediction of its efficacy [29]. Besides theses indications, bullous pemphigoid with eosinophilia [30] and NEAE [31] also showed a good response to reslizumab.

## Conclusions

AE appears to have occurred after COVID-19 vaccination. Reslizumab would be a treatment option for patients with AE who are refractory to systemic steroid therapy.

## Data Availability

All data generated or analyzed during this study are included in this published article.
